# Study of the Electron-Phonon Coupling in PbS/MnTe Quantum Dots Based on Temperature-Dependent Photoluminescence

**DOI:** 10.3390/mi13030443

**Published:** 2022-03-15

**Authors:** Nur Diyana Halim, Muhammad Safwan Zaini, Zainal Abidin Talib, Josephine Ying Chyi Liew, Mazliana Ahmad Kamarudin

**Affiliations:** 1 Department of Physics, Faculty of Science, Universiti Putra Malaysia, Serdang 43400, Selangor Darul Ehsan, Malaysia; diyanahalim88@gmail.com (N.D.H.); safwanzaini44@gmail.com (M.S.Z.); josephine@upm.edu.my (J.Y.C.L.); 2 Department of Physics, College of Natural Sciences, Jeonbuk National University, 567, Baekje-daero, Deokjin-gu, Jeonju-si 54896, Jeollabuk-do, Korea; zainalat@jbnu.ac.kr; 3 Institute of Advanced Technology, Universiti Putra Malaysia, Serdang 43400, Selangor Darul Ehsan, Malaysia

**Keywords:** electron–phonon coupling, confinement, photoluminescence, quantum dots

## Abstract

The temperature dependence of photoluminescence (PL) emission is a valuable tool for investigating carrier localization, recombination, and carrier–phonon interactions. Herein, electron–phonon couplings in lead sulfide (PbS) quantum dots (QDs) and lead sulfide/manganese tellurite (PbS/MnTe) QDs is reported. The effect of temperature on the PL emission of PbS and PbS/MnTe was explored within a temperature range of 10 to 300 K. When temperature increased, PL emission was blue-shifted due to the confinement effect. The gradual broadening of the full width at half maximum (FWHM) with increasing temperature indicates electron–phonon interactions. An analysis based on the Boson model revealed that the values of the exciton acoustic phonon coupling coefficient, σ, and temperature-dependent linewidth, γ, for PbS/MnTe were larger than those for PbS, indicating stronger exciton longitudinal-optical–phonon coupling in the compound structure.

## 1. Introduction

In recent decades, semiconductor quantum dots (QDs) have attracted considerable attention from researchers because of their enormous size-dependent electronic and optical properties [1]. Due to their unique properties such as photostability [2] and bright and narrow photoluminescence (PL) [3,4], QDs are attractive candidates for many future applications such as bioimaging [5], photovoltaics [6], biosensors [7], and photodetectors [8].

Lead sulfide (PbS) QDs have been studied by many researchers due to their narrow bandgap of 0.41 eV and large exciton Bohr radius of ~18 nm, which provide strong quantum confinement effects in large nanocrystals compared with CdS, which has a small Bohr exciton radius of ~6 nm [9,10]. In addition, near-infrared (NIR) PbS QDs have emerged as a promising tool for in vivo deep-tissue imaging applications [11], NIR optoelectronics [12,13], and solar cells [14]. However, QDs possess a high surface-to-volume ratio, making them prone to surface defect/trap formation, which acts as a non-radiative recombination center within QDs, thereby reducing the confinement effect and material stability [15]. This problem can be solved by stabilizing QDs by chemical modification and optimizing the fluorescence of the QD core [16].

By providing effective surface passivation, the shell layer may function as a barrier to protect QD cores from oxidation [17]. Furthermore, by utilizing a larger bandgap material for the shell, the charge carrier may be confined in the core region and protected from surface interactions and the surrounding environment [18,19]. Thus, core/shell QDs will enhance stability against photodegradation and reduce the number of surface dangling bonds [20,21]. Manganese telluride (MnTe) with a bandgap of MnTe 1.3 eV is ideal for this purpose [22]. PbS/MnTe belongs to type-I QDs where the electrons and holes are confined in the core region, thus increasing the confinement energy of QDs, which is useful for applications such as solar cells [23,24]. To date, there have been no reports on the behavior of PbS mixed with MnTe.

This study focuses on the influence of the PbS mixed with MnTe on the optical properties of QDs. The characteristics of PbS and PbS/MnTe QDs PL emission were assessed in the temperature range of 10 K to 300 K. This study also discusses the morphological characteristics of PbS and PbS/MnTe QDs fabricated via colloidal synthesis. 

## 2. Materials and Methods

### 2.1. Materials

1-thioglycerol (TGL, 95%) and dithioglycerol (DTG, 95%) were purchased from Sigma Aldrich (Irvine, UK). Lead (II) acetate trihydrate (Pb(CH_3_COO)_2_.3H_2_O, 99.99%), sodium sulfide nonahydrate (Na_2_S·9H_2_O, 99.99%), manganese (II) acetate hydrate (Mn(CH_3_CO_2_)_2_·4H_2_O, 98%), and hydrazine hydrate (N_2_H_4_, 80%) were obtained from R&M Chemicals (London, UK). Triethylamine (99.5%) was purchased from Chemiz (Shah Alam, Malaysia) and sodium tellurite (Na_2_TeO_3_, 95%) was purchased from Aladdin (Shanghai, China). Deionized water was used throughout the experiments, and all chemicals were used without further purification.

### 2.2. Synthesis of Quantum Dots

PbS QDs were synthesized in an aqueous solution following the procedure as reported in a previous work [25]. Briefly, Pb(CH_3_COO)_2_.3H_2_O (0.190 g), TGL (26 µL), and DTG (10 µL) were dissolved in deionized water, and the mixture was stirred for 15 min under the flow of nitrogen (N_2_) gas. Then, the pH of the solution was adjusted to ~10 by the dropwise addition of triethylamine. Subsequently, the S^2−^ precursor was prepared by dissolving Na_2_S·9H_2_O in deionized water. Next, Pb^2+^ and S^2−^ precursors were mixed and stirred for at least 30 min to form a PbS QDs solution. The molar ratio of Pb^2+^ to S^2−^ was 1:0.3. Meanwhile, the precursor of MnTe was prepared by dissolving 0.060 g of Mn(CH_3_CO_2_)_2_·4H_2_O, 0.032 g Na_2_TeO_3_, and 0.05 g N_2_H_4_ in deionized water. The molar ratio of Mn^2+^ to Te^2−^ was fixed at 1:1. Subsequently, the MnTe precursor was added to the PbS solution and stirred for 30 min under the flow of N_2_.

### 2.3. Sample Characterisation

The PL measurements were carried out using a customized setup equipped with a 532 nm diode-pumped solid-state laser (CNI Laser, Changchun, China), double monochromator (Horiba, Kyoto, Japan), a lock-in amplifier (Stanford Research Systems, Sunnyvale, CA, USA), a chopper (Stanford Research System, Sunnyvale, CA, USA), an InGaAs detector (Horiba, Kyoto, Japan), and temperature controller (LakeShore, Westerville, OH, USA). The temperature-dependent PL was measured between 10 and 300 K in a closed-cycle He cryostat system. For PL measurements, the samples were drop-cast onto a glass substrate. High-resolution transmission electron microscopy (HRTEM) and energy-dispersive X-ray spectroscopy (EDX) were employed for structural characterization. The diluted PbS QDs and PbS/MnTe QDs were drop-cast onto a Cu grid and then covered with an ultrathin-coated film. HRTEM was used to determine the size distribution, shape, and particle size of the QDs in the sample, and EDX was used to determine the elemental composition of the samples.

## 3. Results and Discussion

### 3.1. High-Resolution Transmission Electron Microscopy (HRTEM)

**Figure 1:**
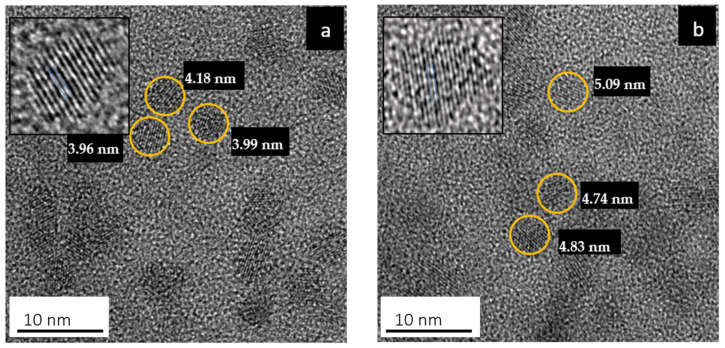
HRTEM images of (**a**) PbS QDs and (**b**) PbS/MnTe compound QDs in an aqueous solution.

Figure 1 shows HRTEM images of the colloidal PbS and PbS/MnTe QDs. Both samples exhibited monodispersed crystalline and spherical particles. As shown in Figure 1a, the average diameter of the PbS QDs is 4.1 ± 0.8 nm. The size enlargement in PbS/MnTe is observed in Figure 1b, with an average diameter of PbS/MnTe being 4.4 ± 0.6 nm. A size increment of ~0.4 nm was expected due to the addition of MnTe. 

### 3.2. Energy Dispersive X-ray (EDX)

**Figure 2:**
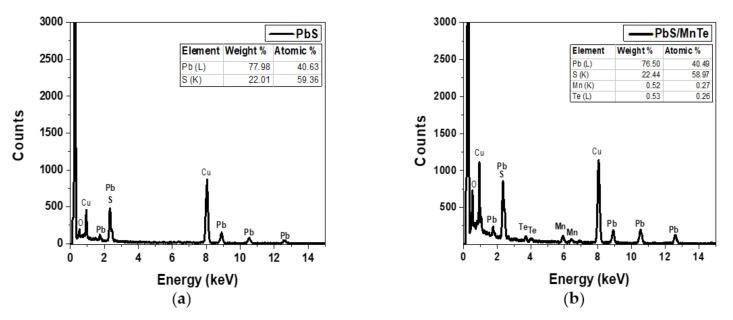
EDX spectrum of (**a**) PbS QDs and (**b**) PbS/MnTe QDs.

The EDX spectra of the PbS and PbS/MnTe samples are presented in Figure 2. The Cu peaks observed in Figure 2a,b correspond to the copper grid used in the HRTEM, whereas the overlapping peaks at 2.3 keV confirm the presence of Pb and S. The atomic percentage ratio between Pb and S is 1:4 is compared to 1:0.3, as mentioned in sample preparation. The higher amount of S, which is four times higher than Pb, might be due to the capping ligands on the surface of PbS QDs (TGL and DTG) that have functional groups of sulfur atoms [26]. Figure 2b supports the existence of Mn and Te, which were observed at 5.9 keV and 3.7 keV, respectively, in addition to Pb and S.

### 3.3. Photoluminescence (PL)

**Figure 3:**
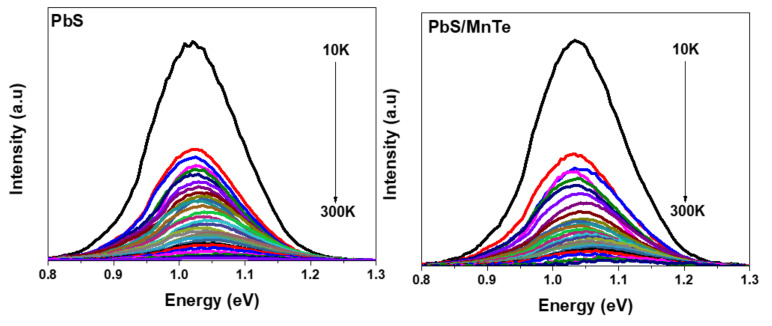
PL spectra of PbS QDs and PbS/MnTe QDs at temperatures between 10 K and 300 K.

**Figure 4:**
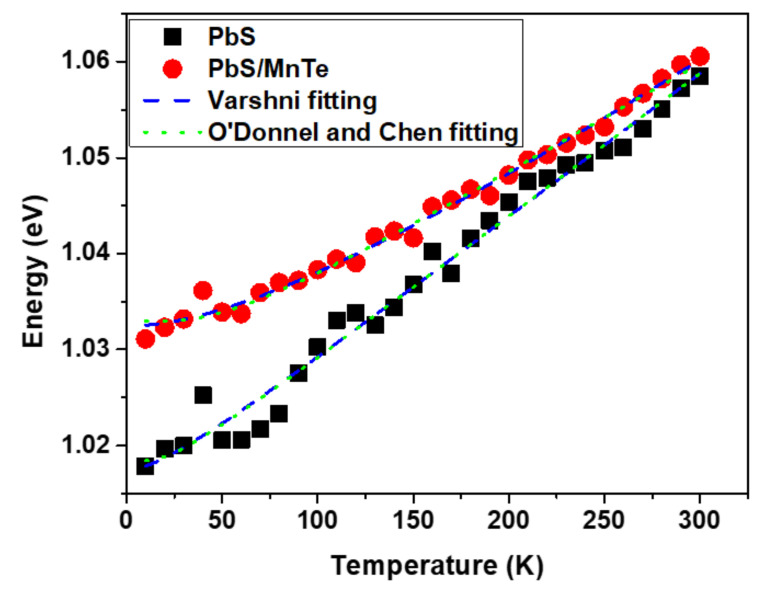
The variation of PL peak energy as a function of the temperature of PbS and PbS/MnTe.

Figure 3 shows the PL spectra of PbS and PbS/MnTe measured at temperatures between 10 and 300 K. Generally, the PL intensity of both samples increases with a decrease in temperature. This is probably due to the electron–phonon coupling [27]. Moreover, the spectral peak blue-shifted with the increase in temperature for both samples, as presented in Figure 4. The blue shift of the PL energy peaks with temperature can be explained by the thermal expansion of the crystal lattice and electron–phonon coupling [28]. The charge carriers at the core of the QDs interact with lattice vibrations through phonons. Because the charge carriers are randomly frozen into QDs states at low temperatures, the PL spectrum represents the distribution of QDs energies. The interaction between the charge carriers and phonons causes a uniform widening of the optical linewidth as the temperature rises. Furthermore, the increase in phonon scattering causes a decrease in PL intensity. The blue shift in the PL peak energy is due to thermal expansion, where strain on the QDs increases as temperature increases.

As observed in Figure 4, the peak energy of the PbS QDs was shifted from 1.018 eV to 1.059 eV. However, in PbS/MnTe, the peak energy shifted from 1.031 to 1.061 eV. This indicates that the presence of MnTe allows the emission spectrum to be tuned to a higher energy. Furthermore, the energy shift in PbS/MnTe is smaller than that in PbS, with an energy shift of ~30 meV probably due to thermal expansion [29]. The thermal expansion coefficient can be explained by fitting data in Figure 4 using Equation (1) as presented by Varshni [30]:

(1) $$E_{g}\left(T\right)=E_{g}\left(0\right)\;+\;\alpha \frac{T^{2}}{\beta +T}$$

where *E_g_*(0) is the bandgap at *T* = 0 K, *α* is the temperature coefficient of the bandgap energy, and *β* is a constant of the order of magnitude of the semiconductor material’s Debye temperature. Equation (1) was modified by changing the negative sign in the second term to the positive sign since the bandgap energy of PbS QDs increases with the increasing temperature, which is in contrast to other materials [25]. Several factors can contribute to the increase in the energy bandgap of the QDs with temperature. The thermal expansions of the crystal lattice and electron–phonon coupling are included [31,32]. Consequently, the strain between the thermal expansion mismatches also causes a change in the energy bandgap of QDs with temperature [33]. The strain on QDs increases as the temperature rises.

The value of the temperature coefficient, *α = dE*/*dT*, can be extracted from the peak energy versus temperature graph in Figure 4. Based on the graph, the value *α* for PbS QDs is 0.15 meV/K, which is smaller than bulk PbS [34] but comparable to other PbS QDs that were reported [32]. However, the value of *α* for the PbS/MnTe compound QDs decreased slightly (*α* = 0.11 meV/K). Generally, the value of *dE*/*dT* is mainly attributed to the thermal expansion coefficient and electron–phonon interactions [35,36]. In specific terms, the value of the thermal expansion coefficient of bulk MnTe (−3.42 meV/K) was smaller than that of PbS in bulk (0.52 meV/K). Consequently, the lower *dE*/*dT* value of the PbS/MnTe compound QDs was caused by the negative value of the thermal expansion coefficient of bulk MnTe. Additionally, the presence of MnTe limits the lattice dilation of the PbS core. In addition, the electron–phonon interactions might also be a reason for the reduction in *dE*/*dT* [34]. The electron–phonon coupling strength in semiconductor nanostructures can be explained by the Huang–Rhys factor as in previous work [37,38]. Figure 4 shows the graph fitted using Equation (2) [39]:

(2) $$E_{g}\left(T\right)=E_{g}\left(0\right)+\frac{2SE_{LO}}{exp\frac{E_{LO}}{k_{B}T}-1}$$

where *E_g_*(0) is the value of the bandgap at 0 K, *S* is the Huang–Rhys factor, *E_LO_* is the average phonon energy, and *k_B_* is the Boltzmann constant. The value of *S* for PbS and PbS/MnTe obtained from the fitting results is 0.4 and 0.7, respectively, while the value of *E_LO_* is 5 meV and 12 meV, respectively. The values obtained are in good agreement with previous work [40]. This increase in value indicates an increase in phonon coupling in the presence of MnTe.

**Figure 5:**
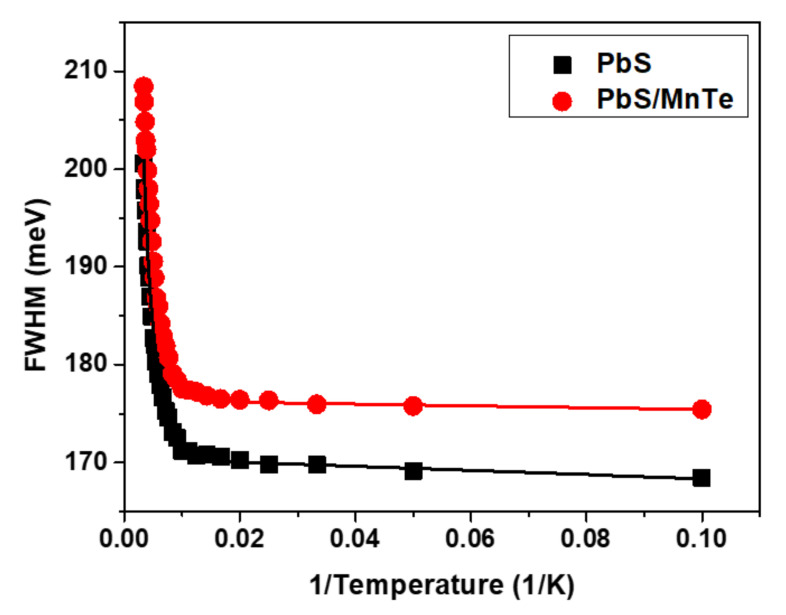
Temperature dependence of the FWHM for PbS and PbS/MnTe.

Figure 5 shows the temperature dependence of FWHM for PbS and PbS/MnTe. Broadening of the FWHM of both samples was observed when the temperature increased from 10 to 300 K due to electron–phonon interactions. The temperature dependence of the FWHM can be represented by the Boson model in Equation (3):

(3) $$\Gamma \left(T\right)=\Gamma_{inh}+\sigma T+\gamma N_{LO}\left(T\right)$$

where *Γ_inh_* is the inhomogeneous broadening term, *σ* is the exciton acoustic phonon coupling coefficient, *γ* is the temperature-dependent linewidth parameter characterizing the total linewidth due to the exciton longitudinal-optical (LO) phonon interaction, and $N_{LO}\left(T\right)=exp\left(E_{LO}/k_{B}T\right)$ is the Bose–Einstein distribution of the LO phonon. All data were fitted by setting $\hbar \omega_{OP}=26\;meV$ equal to the energy of LO phonons in PbS [41]. The parameters of *Γ_inh_, σ*, and *γ* from Equation (2) for PbS and PbS/MnTe are listed in Table 1.

**Figure 6:**
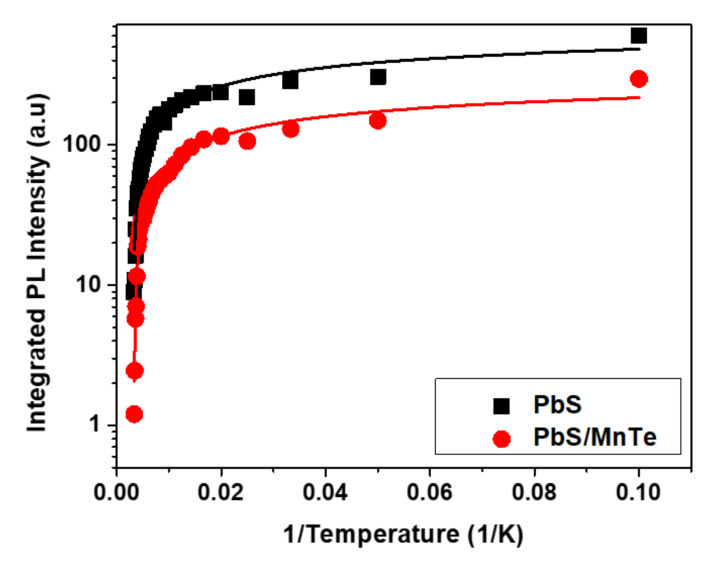
Temperature dependence of PL intensity for PbS and PbS/MnTe.

The value of *Γ_inh_* is not related to temperature because of inhomogeneous broadening, which might be due to the fluctuations in the size, shape, and elemental composition of QDs [42,43]. Interestingly, the values of *σ* and *γ* for PbS/MnTe were larger than those for PbS, indicating stronger exciton LO–phonon coupling [44] in these systems. Moreover, the higher values of *σ* and *γ* for PbS/MnTe compared with PbS are defined by noticeably stronger quantum confinement, as previously observed [45]. Furthermore, for PL, the decrease in PL peak intensity with temperature can be observed in Figure 6. The decreasing intensity is related to the excitation of carriers from the QDs into non-radiative recombination centers or Auger recombination. To understand the role of recombination, the intensities of the PL spectra of PbS and PbS/MnTe were fitted using the Arrhenius equation as in Equation (4):

(4) $$I\left(T\right)=\frac{I_{0}}{1+Ae^{\frac{-Ea}{K_{b}T}}}$$

where *I_0_* is the PL intensity at 0 K, *A* is the fitting coefficient, *E_a_* is the activation energy of the thermal quenching, and *K_b_* is the Boltzmann constant. The value of *E_a_*, which was obtained from the fitting graph in Figure 6 for PbS and PbS/MnTe compound QDs, are 11.12 ± 0.61 meV and 5.51 ± 0.29 meV, respectively. The value of the thermal activation energy is influenced by the exciton binding energy [46], potential barrier [47], surface state/trap [34], and electron–phonon interaction [48]. The values of *E_a_*, which were obtained from the fitting graph in Figure 6 for PbS and PbS/MnTe compound QDs, are 11.12 ± 0.61 meV and 5.51 ± 0.29 meV, respectively. The thermal activation energy is influenced by the exciton binding energy [37], potential barrier [38], surface state/trap [31], and electron–phonon interactions [39]. The presence of the potential barrier MnTe had modified the band energy where the carriers are trapped at the center of the core region. This strongly affects the Coulombic interaction and, thus, affects the charge carrier transport.

The PL intensity showed a slight decrease with increasing temperature for both PbS and PbS/MnTe QDs. The decrease in intensity with temperature is the cause of the significant non-radiative carrier relaxation channels in the semiconductors [49,50]. At high temperatures, phonon coupling is strong, and the nonradiative rate becomes high and, as a result, the PL spectra become more sensitive to the temperature.

## 4. Conclusions

In summary, we investigated PL temperature dependence of PbS and PbS/MnTe QDs at temperatures ranging from 10 to 300 K. According to these findings, increasing the temperature had affected PL peak energy, FWHM, and intensity of both the samples. The PL peak energies of both samples were blue-shifted with increasing temperatures because of the increasing distance between atoms in QDs as temperature increased. Furthermore, the value of *dE*/*dT* for the PbS/MnTe was smaller than that of the PbS core QDs due to the crystal dilation and electron–phonon interaction. Based on FWHM analysis, it can be concluded that PbS/MnTe QDs have stronger LO–phonon interactions than bare PbS QDs due to the greater values of *σ* and *γ*, and this reveals that compound QDs have strong quantum confinement effects.

## Figures and Tables

**Table 1:** Parameters obtained from Equation (2) that have been fitted as in Figure 5.

Samples	$\Gamma_{inh}\;\left(meV\right)$	$\sigma \;\left(\mu eV\;K^{-1}\right)$	$\gamma \;\left(meV\right)$
PbS	168.94 ± 0.64	113.93 ± 1.54	89.33 ± 1.06
PbS/MnTe	174.86 ± 0.58	117.53 ± 1.63	91.16 ± 1.17
